# Exploring the complex mechanical properties of xanthan scaffolds by AFM-based force spectroscopy

**DOI:** 10.3762/bjnano.5.42

**Published:** 2014-03-27

**Authors:** Hao Liang, Guanghong Zeng, Yinli Li, Shuai Zhang, Huiling Zhao, Lijun Guo, Bo Liu, Mingdong Dong

**Affiliations:** 1 Institute of Photo-biophysics, School of Physics and Electronics, Henan University, Kaifeng, 475004 Henan, PR China; 2 Interdisciplinary Nanoscience Center (iNANO), Aarhus University, DK-8000 Aarhus, Denmark

**Keywords:** atomic force microscopy (AFM), force spectroscopy (FS), mechanical properties, xanthan scaffold

## Abstract

The polysaccharide xanthan has been extensively studied owing to its potential application in tissue engineering. In this paper, xanthan scaffold structures were investigated by atomic force microscope (AFM) in liquid, and the mechanical properties of the complex xanthan structures were investigated by using AFM-based force spectroscopy (FS). In this work, three types of structures in the xanthan scaffold were identified based on three types of FS stretching events. The fact that the complex force responses are the combinations of different types of stretching events suggests complicated intermolecular interactions among xanthan fibrils. The results provide crucial information to understand the structures and mechanical properties of the xanthan scaffold.

## Introduction

In general, a scaffold is composed of small units including sheet-like, cylinder-like, tube-like, sphere-like and sponge-like structures. Scaffold structures formed by various biopolymers have attracted more and more attention due to their potential applications in tissue engineering [1], such as cell incubation [2] and the repair of damaged tissue [3]. Xanthan, a polysaccharide which can self-associate into a scaffold structure [4–5], has been widely used in various fields, such as food additives [6] and drug delivery [7–8].

A number of tools, including NMR [9–10], circular dichroism (CD) [11], and atomic force microscopy (AFM) [12–14], has been used to explore the structures and properties of biopolymer scaffolds. Owing to its high resolution and versatility, AFM stands out of various tools and has been extensively employed in the study of biomaterials. For example, various morphologies of xanthan-based materials, such as fibrils, networks [4] and ring-like structures [5], have been revealed by AFM imaging. Furthermore, AFM is a powerful tool for studying the mechanical properties on the nanoscale. AFM-based force spectroscopy (FS) has been applied to investigate the fingerprint mechanical properties of single molecules [15–16]. FS was firstly used to study the polysaccharide dextran [17], and was later extended to other molecules such as DNA [18–19], proteins [20–21], other polysaccharides [22–24], and amyloid proteins [25–26]. Mechanical properties such as tensile strength, adhesive properties, and elastic modulus [27–29], have been investigated by FS. In the mechanical measurements of biomolecules, the unfolding of the regular secondary structure of proteins was characterized by periodical peaks on the force–distance curves, which allowed for the identification of the rupture force and the characteristic separation distance in the proteins [30]. In addition, the investigation of the mechanical properties of denatured and native polysaccharides such as xanthan fibrils has been carried out carefully [31]. Force plateaus were observed during the stretching of native xanthan, which could be attributed to the transition of helical secondary structures. In contrast, no plateaus were found during the stretching of denatured xanthan, which had no ordered secondary structures. Govedarica et al. [32] also concluded that the radius of gyration and the persistence length were responsible for the macroscopic polymer behavior. Therefore, it is very important to investigate the mechanical response of polymer complexes after manipulation.

In this study, the morphologies and mechanical properties of complex xanthan scaffolds, a new nanomaterial, were investigated by AFM and FS, respectively. The xanthan scaffold structures were obtained at both air/mica and isopropanol/mica interfaces, and three representative structures were probed by FS. We used a straightforward method to explain the complex force curve of the xanthan scaffold structure. The complex mechanical responses are actually the combinations of the force curves of three representative structures. Besides, the relative small persistence length in our study could indicate that xanthan is in its denatured form in isopropanol under our experimental conditions, which is of great importance in understanding the mechanical properties of xanthan scaffold material.

## Experimental

Xanthan powder (Sigma-Aldrich Co.) was fully dissolved into deionized water by magnetic stirring of 24 h to prepare a 10 g/L stock solution, which then was annealed at 60 °C for 6 h and cooled to room temperature to obtain the xanthan scaffold solution [33]. The annealed 10 g/L xanthan stock solution was diluted to 0.01 g/L for further use. Two different surface adsorption methods were employed in our experiments. For AFM imaging under ambient conditions, 2 μL xanthan solution was dropped onto a freshly cleaved mica substrate (Ted Pella, Inc.) and air-dried for about 30–60 min. For AFM imaging in liquid and force spectroscopy measurements, 2 μL xanthan solution was deposited onto mica. After 60 s adsorption, an O-ring cell was equipped and a suitable amount of isopropanol (J&K Co) was injected as imaging buffer [5]. The AFM experiments were performed after 15 min of stabilization.

### Atomic force microscopy

**AFM imaging:** AFM measurements were conducted on a commercial Agilent AFM/STM 5500 microscope (Agilent Technologies, USA) in contact mode. Nitride silicon cantilevers (OMCL-TR400PSA-1) with a spring constant of 0.02 N/m and a nominal tip radius of approximately 15 nm was used. The experiments were carried out under ultra-clean conditions at room temperature, and AFM imaging was performed both in air and isopropanol with a scanning frequency of 1 Hz and a vertical deflection of 0.5 V was applied. All the AFM images with 512 × 512 pixels were obtained at separate locations to ensure a high degree of reproducibility of experiment data. The images and force data were analyzed by the commercial software Scanning Probe Image Processor (SPIP^TM^, by Image Metrology ApS, version 5.1.3, Lyngby, Denmark).

**Force spectroscopy:** Mechanical measurements of the xanthan scaffolds were performed by force measurement at a loading rate of 1 µm/s. The FS experiments were performed in isopropanol as buffer [5] in neutral environment with the diluted solution. The measurement started with the tip approaching the sample surface until a predefined deflection value was reached. The tip was then retracted from the surface and returned to its initial position. During the process, a force pulling curve was recorded. If the tip picked up xanthan fibrils on the surface, the fibrils would be stretched before they tear off from the tip. Depending on the number of attachment points, at which xanthan fibrils were attached to the tip, single or multiple rupture events may be observed in a single stretching.

## Results and Discussion

**Figure 1:**
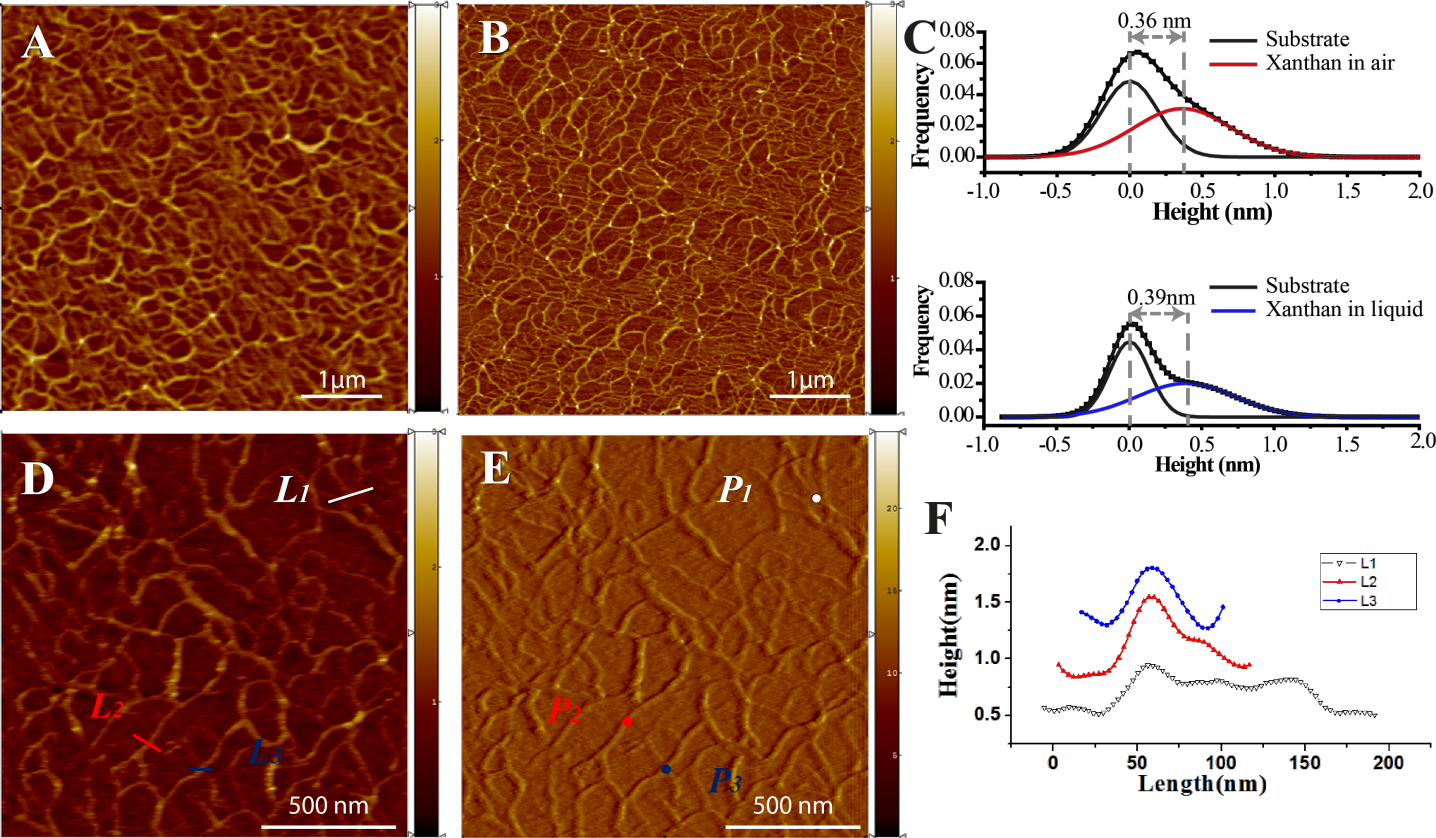
AFM images of xanthan scaffold A) in air, B) in isopropanol. C) Height histograms of xanthan scaffold in air and in isopropanol, respectively. D) Topography image in isopropanol. E) Corresponding deflection image in isopropanol. F) Line profiles that correspond to the marks L_1_, L_2_ and L_3_ in Figure 1D.

The physical structure of xanthan molecules both in solid state and in solution is dominated by semi-flexible double helices, which resemble networks of rods linked by junction zones [34]. Multiple rods randomly wind and overlap with each other, forming complex scaffold structures. The contact mode AFM image (Figure 1A) obtained in air reveals the uniformly-spread xanthan scaffold. The AFM image (Figure 1B) in liquid shows a similar yet clearer network structure. Gaussian distributions were applied to fit the height distributions for the images in air and in liquid, respectively. Two populations were found for both samples (Figure 1C), which represent the measured heights of the substrate and the heights of the xanthan scaffold, respectively. The substrate peaks are normalized to 0 nm. Hence, the heights of fibrils in air and isopropanol are around 0.36 nm and 0.39 nm, respectively (Figure 1C). The AFM topography and deflection images (Figure 1D,E) of the xanthan scaffolds were obtained in isopropanol buffer. Three representative structures (Figure 1E, P_1_, P_2_ and P_3_) are identified to perform the mechanical measurements by FS. P_1_ is characterized by multiple overlapping fibrils; P_2_ is characterized by two overlapping fibrils; and P_3_ is characterized by a single-fibril structure. Line profiles of the typical structures are showed in Figure 1F.

**Figure 2:**
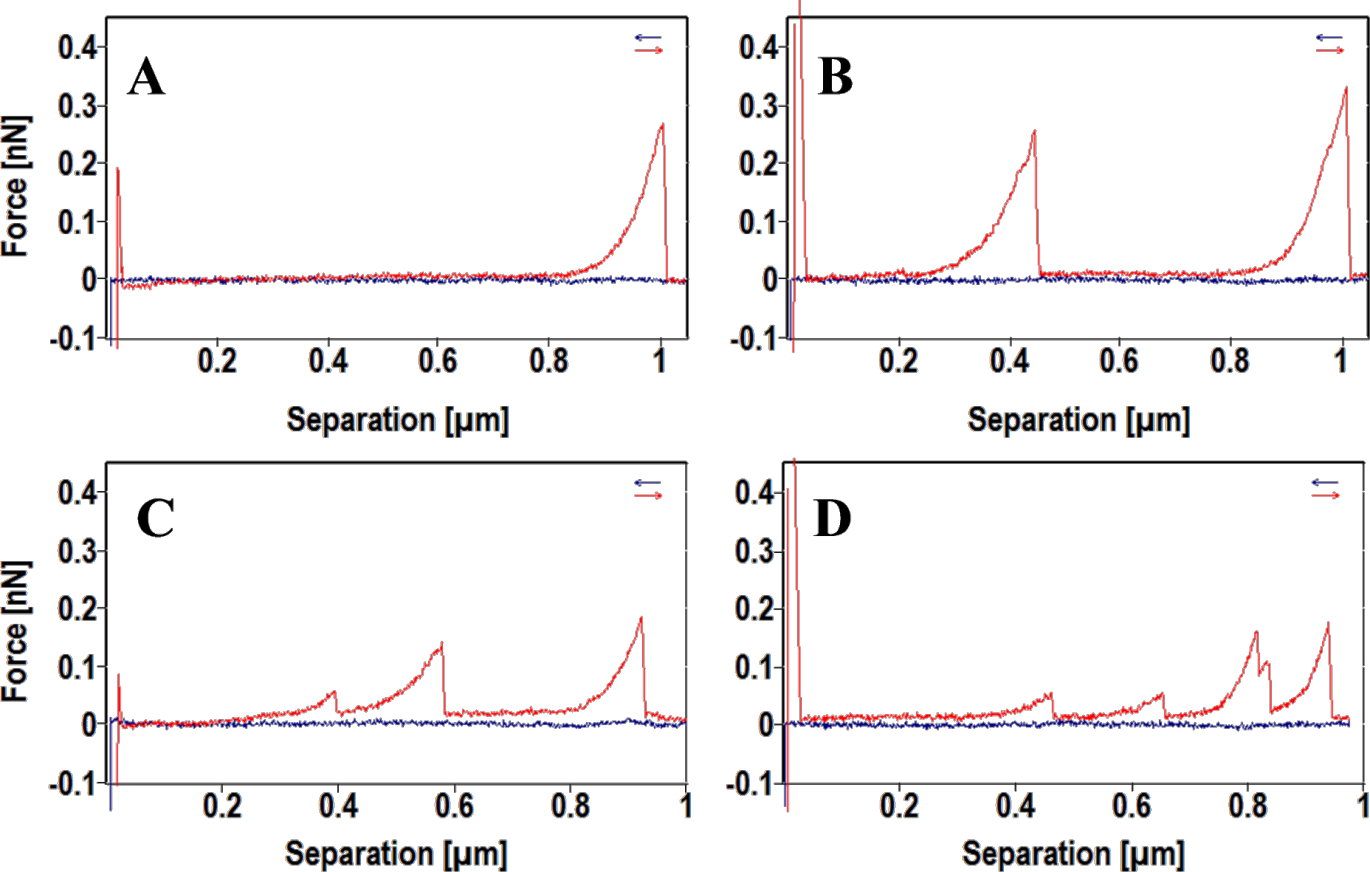
Typical force curves with different number of rupture events. A) Single event. B) Double events. C) Triple events. D) Multiple events.

The above results confirmed our previous morphological studies on the temperature-enhanced re-organization of xanthan gels into 2D network of fibers. Based on this, we move forward to investigate the mechanical properties of the scaffolds by FS. FS was carried out on the xanthan scaffolds in isopropanol buffer, and four typical kinds of force curves with different numbers of rupture events were obtained (Figure 2). A single event curve (Figure 2A) is characterized by a single peak with a large rupture force, which indicates the stretching and rupture of a single xanthan fibril. Double and triple events curves (Figure 2B and Figure 2C) are characterized by two or three independent peaks, which indicate that the AFM tip fished two or three xanthan fibrils at the same time. Multiple events are usually observed during the manipulation at point P_1_ (Figure 2D), indicating that more than three fibrils were attached, yielding sequential ruptures and intermolecular interactions.

**Figure 3:**
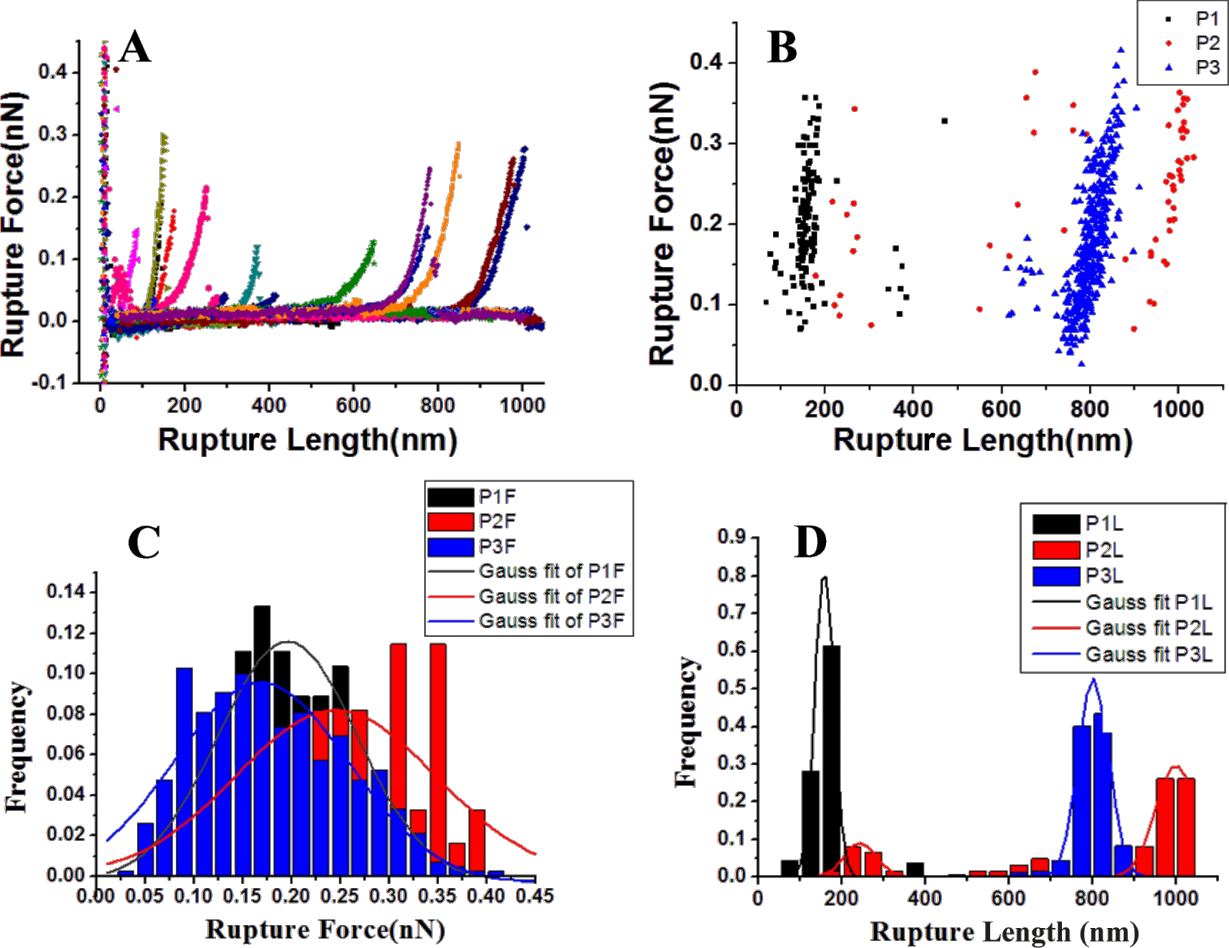
A) Schematic diagram of the superposition of force curves with single events. B) Distributions of rupture length and rupture force of various single events obtained by manipulating distinctive structures P_1_, P_2_ and P_3_. C,D) The frequency distributions of rupture force and rupture length.

Force curves with one peak could be obtained during manipulating all three typical structures. The peak corresponds to a single stretching event. For convenience, this kind of curve is defined as “type 1” (t_1_), to distinguish it from the more complicated force curves, which will be discussed later. The schematic diagram in Figure 3A shows a superposition of 13 force curves with single events but with different rupture lengths, which range from tens to thousands of nanometers. Figure 3B shows a distribution of single events with different rupture forces and rupture lengths obtained by manipulating three different structures. The rupture force is the force needed to break the interaction between the xanthan fibrils and the AFM tip, and the rupture length represents the length of fibrils being stretched. Three domains exhibit distinctive trends of mechanical response. For clearer comparison, the distributions of rupture force and rupture length were separately illustrated in two histogram schematics (Figure 3C and Figure 3D). The distributions of rupture force that were obtained by pulling the three different structures show a similar trend, although the rupture length distributions are distinctive for each measurment. The rupture forces (Figure 3C) range from 50 to 400 pN, indicating the value of the nonspecific interaction force between the AFM tip and a single xanthan fibril. However, the rupture lengths of the force curves in manipulating three different structures are different. At P_1_, the rupture length is small, ranging from 50 to 200 nm. In contrast, the rupture length at P_3_ is much larger, ranging from 750 to 900 nm. The huge difference of rupture length can be attributed to the different length of free xanthan fibrils between the junctions in the fibril network. At P_1_, xanthan fibrils intensely wind and overlap with each other, which results in shorter free fibrils and thus much smaller rupture lengths than those of P_3_, at which the single fibril is attached to two fibrils far away from each other. At P_2_, the rupture length is similar to that at P_3_. The distribution is slightly wider, which is possibly the result of the tip picking up the underlying fibril at P_2_.

**Figure 4:**
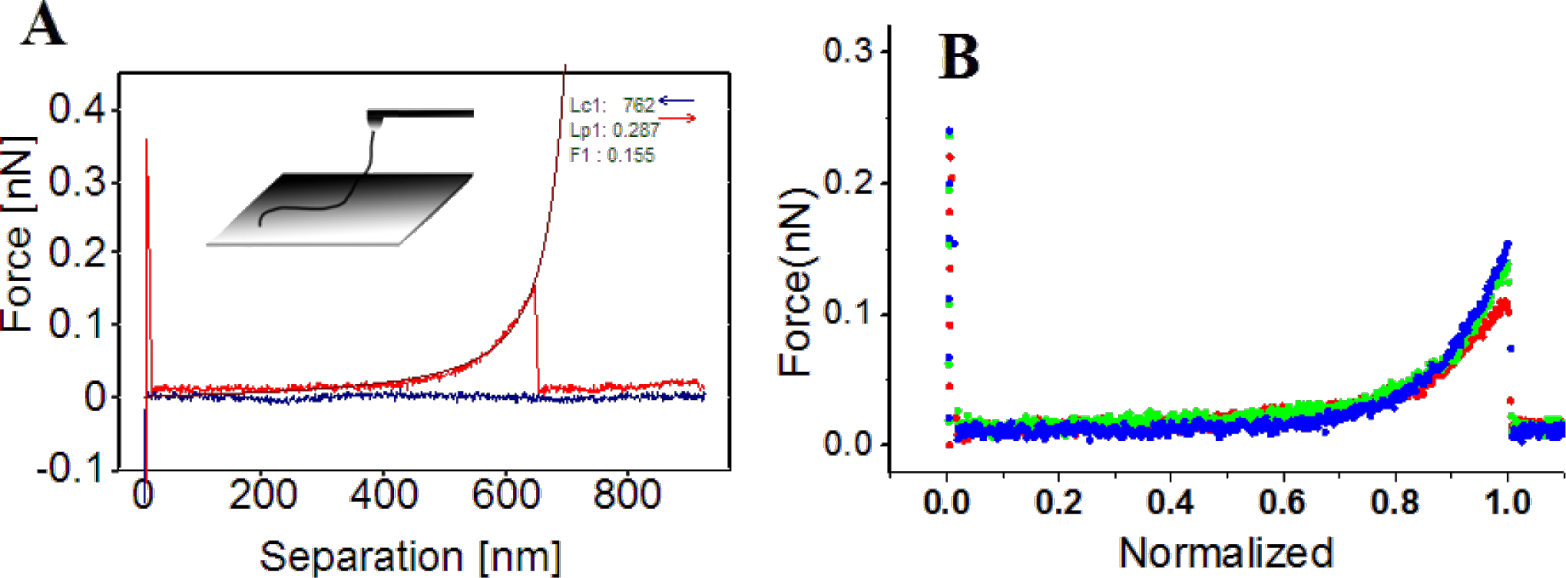
A) Typical “type 1” (t_1_) force curve fitted with the WLC model. The inset is the model proposed to illustrate the single stretching events. B) The superposition of normalized single events (*n* = 3).

In addition to rupture force and rupture length, other information, such as the molecular elasticity, can be derived from the force curves by fitting the force pulling peaks with proper models. The worm-like chain (WLC) [35] model is usually applied to study the behavior of semi-flexible polymers. The equation is as follows:


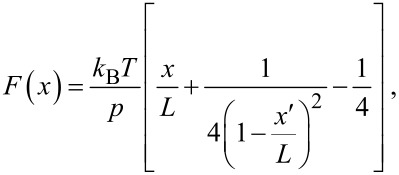


where the contour length, *L*, represents the length of the lifted fibrils and the persistence length, *p*, is a parameter for describing the flexibility of polymer coils, which is defined by the decay length of the directional correlation function along the polymer chain [26]. As showed in Figure 4A, the single stretching event observed at P_3_ was well fitted by WLC model. The superposition of typical force curves after normalization of the separation length indicated that the force curves were measured from identical fibers. The persistence length is 0.35 ± 0.27 nm and contour length is 954 ± 157 nm (*n* = 92). It should be noted that the measured persistence length exhibits a much smaller value than that in the previous study [36–37]. Actually, the stiffness of a polymer depends on the specific experimental environment, e.g., ionic strength, salt concentration, and solution pH can largely influence the measured persistence length of xanthan [32,38–40]. In our study, we used isopropanol instead of water. Xanthan likely forms more hydrogen bonds in water than in isopropanol, and therefore the stiffness measured in isopropanol should be less than that in water. The discrepancy of measured persistence length between our study and the previous study could also suggest that the helical structure of xanthan may collapse and that the xanthan in the scaffold is denatured in isopropanol under our experimental conditions, which can weaken the stiffness of xanthan.

**Figure 5:**
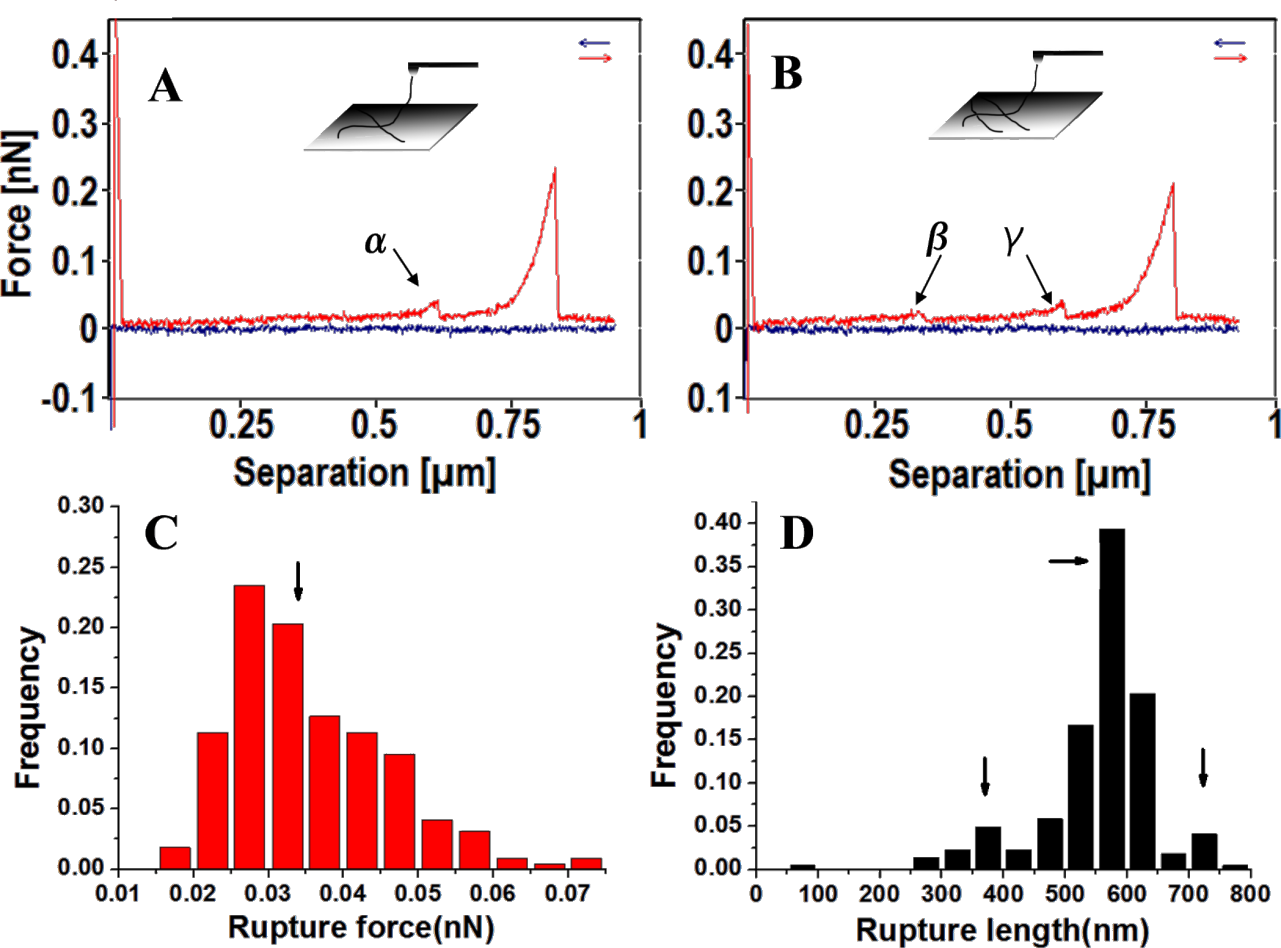
A,B) Typical single stretching event with one and two kinks (type 2), the insets are the proposed models. C,D) The frequency distributions of rupture force and rupture length of kinks in force curves with only one kink, respectively.

Apart from single large peaks, much smaller peaks were observed preceding the large peaks, as shown in Figure 5A and Figure 5B, which indicated that the pulled fibrils experienced one (arrow α in Figure 5A) or more tiny mechanical responses (arrow β and γ in Figure 5B) before the rupture from the AFM tip. These force curves are defined as “type 2” (t_2_). Type 2 force curves frequently occurred during the stretching of the xanthan scaffold. The rupture force of a single kink is around 33 pN (arrow in Figure 5C), and the rupture length is mostly between 450 and 650 nm (arrow in Figure 5D). For force curves with two kinks, the rupture force distribution is similar to that of a single kink. However, a minority concentrates at 175 pN (data not shown), which can be contributed to the weight of fibrils attached on tip. The rupture length distribution agrees well with the dimension of the scaffold cavity.

**Figure 6:**
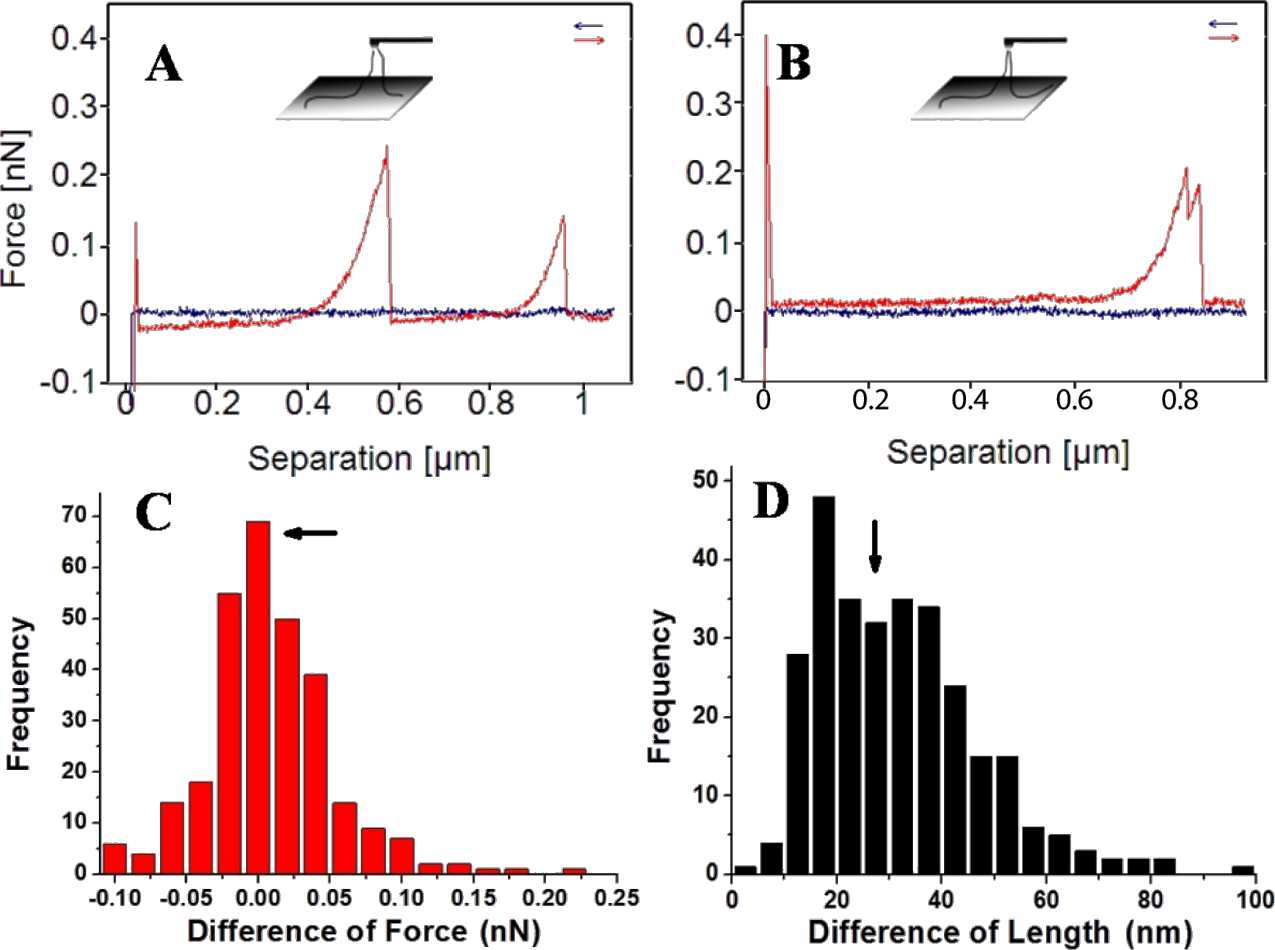
A) Typical double-peak force curve. B) “type 3” (t_3_) force curve. C,D) histograms of the differences between the rupture forces and rupture lengths of the two continues peaks in t_3_ force curves.

The network structure is composed of randomly winding fibrils. Usually, more than two fibrils could be picked up at the same time during the manipulation. Figure 6A shows a typical double-event force curve composed of two independent single peaks. This kind of force curve was mainly obtained in manipulating the structures P_1_ and P_2_. The inset is a proposed model (in Figure 6A), in which two fibrils with different lengths were simultaneously pulled away from mica substrate. The shorter one ruptured from the tip first, followed by the longer one. Another type of double-event force curves (Figure 6B) was frequently observed, which is characterized by two continuous peaks, i.e. the second peak rises before the first peak falls back to zero. This type of force curves is defined as “type 3” (t_3_) force curves. The corresponding mechanism might be explained in the way that two xanthan fibrils were pulled away from mica and ruptured from the AFM tip almost simultaneously. By further analysis of the FS data, it is found that the difference of the rupture forces of the two independent peaks (as in Figure 6A) is around 80 pN (not shown in the plot). One possible reason is that the weight of the detached fibrils contributes to larger rupture force of the second peak. In contrast, the difference between the rupture forces of the two continuous peaks (like Figure 6B) is around 0 nN (arrow in Figure 6C), as the two continuous peaks ruptured from the AFM tip almost at the same time. The rupture length difference between the two peaks is about 30 nm (arrow in Figure 6D), which is comparable with the dimension of the AFM tip, suggesting the two fibrils might be attached at both sides of the tip.

**Figure 7:**
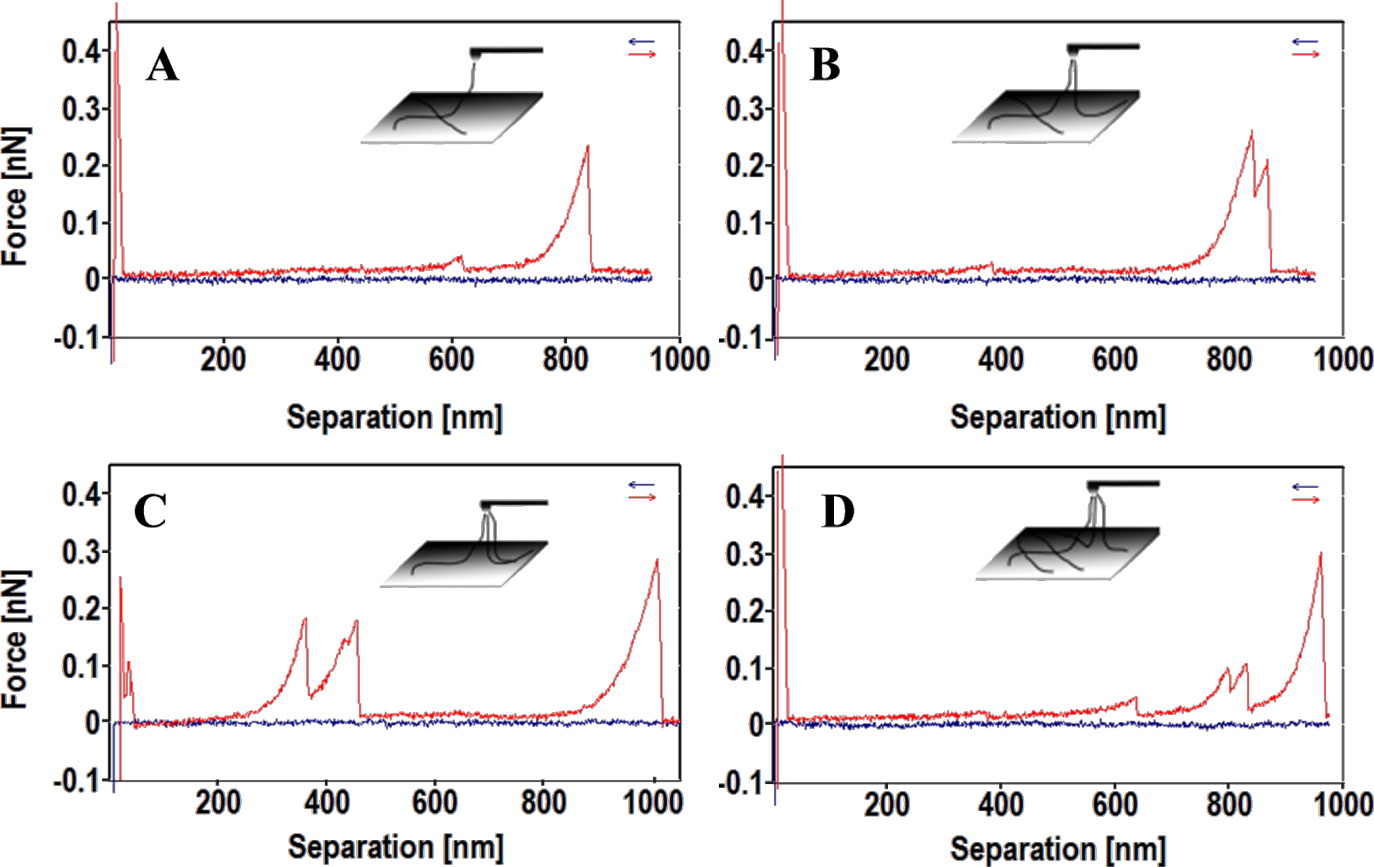
Mechanical responses composited by different type force curves. A) t_1_ + t_2_. B) t_2_ + t_3_. C) t_1_ + t_3_. D) t_2_ + t_2_ + t_1_ + t_3_.

More complex mechanical responses were observed which can be deconvoluted into three typical force events. Figure 7A shows a combination of a t_2_ and a t_1_ force event, Figure 7B is composed of a t_2_ and a t_3_ force event, Figure 7C is composed of a t_3_ and a t_1_ event, and Figure 7D is composed of two t_2_, one t_3_ and one t_1_ event. The insets are the models proposed to interpret the complex mechanical responses. As is shown in the inset of Figure 7A, the pulled fibril was detached from the underlying fibril before rupturing from the AFM tip. The tiny mechanical response is due to the adhesion force between the overlapping fibrils. As a more complex example, the inset of Figure 7B illustrates the case in which the tip fished two fibrils, one of which detached from a third underlying fibril before the two fibrils ruptured from the AFM tip. The force curve in Figure 7C is even more complex in that three fibrils were attached on the tip. Two of them ruptured first, followed by the longest third one. Whereas the most complex force curve is shown in Figure 7D. Similar but different from the one in 7C, the three fibrils experienced two fibril–fibril detachments before they ruptured from the tip sequentially. However, whether one of the fibrils experienced two detachments or two of the fibrils experienced one detachment independently cannot be distinguished from the force curve.

## Conclusion

Scaffold structures of xanthan molecules were studied by AFM under ambient and liquid conditions. After AFM imaging in liquid, the mechanical properties of the xanthan scaffold were explored with force spectroscopy. Among various force responses observed, three basic types of force curve patterns were observed. Type 1 is characterized by a large peak indicating a single fibril was pulled away from mica substrate. Type 2 is characterized by a tiny peak corresponding to the separation of two overlapping fibrils. The rupture force of around 33 pN is the interaction force between two fibrils. Type 3 is characterized by two continuous peaks suggesting that two fibrils were attached on the AFM tip and ruptured almost at the same time. More complex force curves were explained by combined models. The investigation of the mechanical properties of xanthan scaffold provides significant information toward understanding the self-assembled xanthan scaffold structure.
